# The nucleotide sequence and genome organization of *Plasmopara halstedii *virus

**DOI:** 10.1186/1743-422X-8-123

**Published:** 2011-03-17

**Authors:** Marion Heller-Dohmen, Jens C Göpfert, Jens Pfannstiel, Otmar Spring

**Affiliations:** 1 Institute of Botany, University of Hohenheim, 70593 Stuttgart, Germany; 2 Department of Plant Pathology, Michigan State University, East Lansing, Michigan, USA; 3 Institute of Physiology, University of Hohenheim, 70593 Stuttgart, Germany

## Abstract

**Background:**

Only very few viruses of Oomycetes have been studied in detail. Isometric virions were found in different isolates of the oomycete *Plasmopara halstedii*, the downy mildew pathogen of sunflower. However, complete nucleotide sequences and data on the genome organization were lacking.

**Methods:**

Viral RNA of different *P. halstedii *isolates was subjected to nucleotide sequencing and analysis of the viral genome. The N-terminal sequence of the viral coat protein was determined using Top-Down MALDI-TOF analysis.

**Results:**

The complete nucleotide sequences of both single-stranded RNA segments (RNA1 and RNA2) were established. RNA1 consisted of 2793 nucleotides (nt) exclusive its 3' poly(A) tract and a single open-reading frame (ORF1) of 2745 nt. ORF1 was framed by a 5' untranslated region (5' UTR) of 18 nt and a 3' untranslated region (3' UTR) of 30 nt. ORF1 contained motifs of RNA-dependent RNA polymerases (RdRp) and showed similarities to RdRp of *Scleropthora macrospora *virus A (SmV A) and viruses within the Nodaviridae family. RNA2 consisted of 1526 nt exclusive its 3' poly(A) tract and a second ORF (ORF2) of 1128 nt. ORF2 coded for the single viral coat protein (CP) and was framed by a 5' UTR of 164 nt and a 3' UTR of 234 nt. The deduced amino acid sequence of ORF2 was verified by nano-LC-ESI-MS/MS experiments. Top-Down MALDI-TOF analysis revealed the N-terminal sequence of the CP. The N-terminal sequence represented a region within ORF2 suggesting a proteolytic processing of the CP *in vivo*. The CP showed similarities to CP of SmV A and viruses within the Tombusviridae family. Fragments of RNA1 (ca. 1.9 kb) and RNA2 (ca. 1.4 kb) were used to analyze the nucleotide sequence variation of virions in different *P. halstedii *isolates. Viral sequence variation was 0.3% or less regardless of their host's pathotypes, the geographical origin and the sensitivity towards the fungicide metalaxyl.

**Conclusions:**

The results showed the presence of a single and new virus type in different *P. halstedii *isolates. Insignificant viral sequence variation indicated that the virus did not account for differences in pathogenicity of the oomycete *P. halstedii*.

## Background

Only very few species within the Oomycetes are known to host virus-like elements such as virus-like particles (VLPs), double-stranded RNA (dsRNA) or single-stranded RNA (ssRNA) (for details see [1]). So far, only the virions of *Sclerophthora macrospora *and *Plasmopara halstedii *have been studied more in detail.

*Sclerophthora macrospora *virus A (SmV A) virus B (SmV B) are the only virions of Oomycetes of which the genome has yet been fully characterized [2,3]. They were isolated from Japanese isolates of *S. macrospora*, the downy mildew pathogen of *Oryza sativa *and other species within the Poaceae family. Both, SmV A and SmV B were isometric and used ssRNA to encode their viral genomes [4,5].SmV B featured one coat protein (CP) of 41 kDa and one ssRNA segment of 5533 nucleotides (nt) encoding two large open-reading frames (ORF). Two CP (43 kDa and 39 kDa) and three segments of ssRNA were found to set up SmV A. RNA1 consisted of 2928 nt and two ORF (ORF1a and ORF1b). ORF1a contained the motifs of the RdRp. The latter showed some similarity in the amino acid sequence to the RdRp of Nodaviridae. RNA2 consisted of 1981 nt and a single ORF (ORF2) which encoded the CP. The CP of SmV A showed similarities to CP of viruses within the Tombusviridae family. RNA3 consisted of 977 nt but no ORF suggesting it as a satellite RNA [3-5].

*P. halstedii *is a worldwide distributed pathogen with a broad spectrum of pathotypes (physiological races) [6], causing sunflower downy mildew infections. Almost 20 years ago, isometric virions were found in a single North American pathotype of *P. halstedii *[7]. The CP was determined to consist of a 37.5 kDa polypeptide and RNA was determined to encode the viral genome [8,9]. A more recent screening of *P. halstedii *isolates from different countries showed the occurrence of morphologically and biochemically indistinguishable virions in all samples independent of their host's origin or pathotype or fungicide tolerance [1].

The virions were isometric and measured approximately 37 nm in diameter. One polypeptide of ca. 36 kDa and two segments of ssRNA (3.0 and 1.6 kb) were detected. Comparison of a partial nucleotide sequence confirmed the uniformity of the virions found in *P. halstedii *isolates. In addition, the deduced amino acid of this RNA fragment indicated similarities to a part of the CP of SmV A [1]. However, final analysis of the relationship of the *Plasmopara halstedii *virus (PhV) to other viruses was constricted due to lack of full genomic data. Here we report on the complete nucleotide sequence and genome organization of PhV and its relationship to other viruses. Additionally we report on the extremely low genetic variation of PhV within several *P. halstedii *isolates of different origin and pathogenicity.

## Methods

### Origin and culturing of the used fungal isolates

*P. halstedii *pathotypes were cultured, characterized, harvested and intermediately stored as described earlier [1]. Isolates of *P. halstedii *used in this study are listed in Table 1. Sporangium samples are deposited in the Oomycetes collection of O. Spring, University of Hohenheim and stored at -70°C. Vouchers of field collections of downy mildew infected sunflower are deposited in the Hohenheim herbarium (HUH) under the collection numbers #405 (Waldenbuch, Germany, 2001), #406 (Wurmlingen, Germany, 2001), #407 (Steinenbronn, Germany, 2001), #530 (Balcarce, Argentina, 2003) and #1057 (Plieningen, Germany, 2008).

**Table 1:** Characteristics of *Plasmopara halstedii *isolates used in this study as well as sequence data for viral RNA1 and RNA2.

*P. halstedii *isolate/viral isolate			Characteristics of *P. halstedii*		Analyzed *P. halstedii *virus sequences			
	**Year of collection**	**Geographic origin**^2^	**Pathotype**	**Metalaxyl^3^**	**RNA1**	**Accession**	**RNA2**	**Accession**

Ph4-93	1993	Fargo, ND, USA	710	*	1921 nt	[GenBank:HM453717]	1446 nt	[GenBank:HM453722]
Ph1-97	1997	Groß Gerau, GER	330	*	*	-	1445 nt	[GenBank:HM453723]
Ph9-98	1998	Leinfelden, GER	310	sensitive	1921 nt	[GenBank:HM453716]	1445 nt	[GenBank:HM453720]
Ph8-99^1^	1999	Bléré, FRA	710	resistant	2793 nt	[GenBank:HM453713]	1526 nt	[GenBank:HM453718]
Ph1-00	2000	Clermond-Ferrand, FRA	703	sensitive	1921 nt	[GenBank:HM453715]	1445 nt	[GenBank:HM453721]
Ph10-00	2000	Poutoux, FRA	710	resistant	*	-	1445 nt	[GenBank:HM453725]
Ph19-01	2001	Gödöllö, HUN	100	*	2430 nt	[GenBank:HM453714]	1445 nt	[GenBank:HM453719]
Ph5-05	2005	Jülich, GER	710	resistant	*	-	1446 nt	[GenBank:HM453724]

* not determined

^1 ^Complete nucleotide sequences were established for this viral isolate.

^2 ^HUN: Hungary, GER: Germany, FRA: France, USA: United States of America

^3 ^*P. halstedii *isolate is either resistant or sensitive to fungicide treatment with metalaxyl [18].

### Virus extraction and purification

Virus extraction was based on former experiments [10] and was empirically adjusted to precipitate adequate amounts of PhV.

At least 180 g of frozen sunflower tissue (primary foliage leaves, cotyledones and stems) infected with different isolates of *P. halstedii *was used to purify virions. Per 1 g infected sunflower tissue, 2 ml 0.05 M sodium phosphate buffer, pH 7.0 were added. Additionally, 0.3% (w/v) of disodium sulfite was added and dissolved by stirring before the sunflower tissue was finely homogenized with an electrical blender. The homogenate was squeezed through four layers of cheesecloth and then centrifuged (5000 g, 4°C, 15 minutes). To the supernatant, 0.5 M sodium chloride was added and dissolved by stirring at room temperature. PEG 6000 was gradually added to a final ratio of 12% (w/v) and the suspension was stirred for an hour before the precipitation was conducted at 4°C for 60 hours. The suspension was centrifuged (7000 g, 4°C, 30 min) and the precipitate was re-suspended in ^1^/_100 _of the original volume of 0.05 M sodium phosphate buffer, pH 7.0 with additional 0.3% (w/v) disodium sulfite. To remove contaminants like ribosomes [11], 20% (v/v) chloroform was added and mixed with the re-suspension. A low-speed centrifugation step (1000 g, 4°C, 5 min) was performed to separate virus-containing aqueous from the organic phase. The chloroform extraction step was repeated once.

Success of the virus extraction and purification procedure was controlled by negative staining for transmission electron microscopy as described earlier [1].

The virus suspension was divided into aliquots and stored at -70°C. Under these conditions, virus suspensions were suitable for RNA extraction, purification and reverse transcription PCR (RT-PCR) experiments for more than three years.

### Protein extraction and mass spectrometry

Extraction of CP of PhV was carried out as described earlier [1]. Samples for nano-LC-ESI-MS analysis were further purified on 10% polyacryl amide gels [12] on a Mini-Protean System (Bio-Rad Laboratories, München, Germany) and stained with coomassie brilliant-blue (Roti-Blue, Carl Roth, Karlsruhe, Germany).

Gel bands of the CP were in-gel-digested using trypsin (Roche, Penzberg, Germany) [13]. After tryptic digestion, the supernatant was recovered and the gel pieces were extracted with 50% acetonitrile (ACN)/50% 0.1% formic acid (FA) (v/v). The pooled supernatants were then dried in a vacuum centrifuge and stored intermediately at -20°C.

Nano-LC-ESI-MS/MS experiments were performed on an ACQUITY nano-UPLC system (Waters, Milford, USA) directly coupled to a LTQ-Orbitrap XL hybrid mass spectrometer (Thermo Fisher, Bremen, Germany). Tryptic digests of the PhV CP were concentrated and desalted on a precolumn (2 cm × 180 μm, Symmetry C18, 5 μm particle size; Waters, Milford, CT) and separated on a 20 cm × 75 μm BEH 130 C18 reversed phase column (1.7 μm particle size; Waters, Milford, CT) using a linear gradient of 1 to 50% ACN in 0.1% FA within 1 h. The LTQ-Orbitrap was operated under the control of XCalibur 2.0.7 software. Survey spectra (m/z = 250-1800) at a resolution of 60.000 at m/z = 400 were detected in the Orbitrap using lock-mass ions from ambient air for internal calibration [14]. Data-dependent tandem mass spectra were generated for the five most abundant peptide precursors in the linear ion trap.

Mascot 2.2 software (Matrix Science, London, UK) was used for protein identification. Spectra were searched against the NCBI protein sequence database downloaded as FASTA-formatted sequences from ftp://ftp.ncbi.nih.gov/blast/db/FASTA/nr.gz and supplemented with the deduced ORF2 amino acid sequence of the PhV CP. Search parameters specified trypsin or "no enzyme" as cleaving enzyme allowing two missed cleavages, a 3 ppm mass tolerance for peptide precursors and 0.6 Da tolerance for fragment ions.

### Characterization of the N-terminus of the CP

Mass spectra of purified PhV CP, in source decay (ISD)- and MS/MS-spectra were acquired on a AutoflexIII MALDI-TOF-TOF mass spectrometer (Bruker Daltonics, Bremen, Germany). The instrument was operated in the positive ion mode and externally calibrated using protein mass or peptide calibration standards (Bruker Daltonics, Bremen, Germany), respectively. PhV CP samples were desalted and concentrated on C4 ZipTips (Millipore, Schwalbach, Germany) following the manufacturer's protocols. Proteins were eluted directly onto a stainless steel target using 1 μl of a 2,5-Dihydroxybenzoic acid matrix solution (30 mg mL^-1 ^in 50% ACN/50% 0.1% TFA, v/v). Molecular masses of intact CP was obtained in the linear mode using an accelerating voltage of 20 kV with 1000 laser shots per sample to ensure good S/N ratio.

ISD-spectra and MS/MS-spectra (T3-Sequencing = terminus-specific pseudo-MS3 TOF-TOF analysis) of PhV CP were acquired in the reflector mode using an accelerating voltage of 20 kV. In order to achieve a good S/N ratio, 3000 and 2000-3000 laser shots were recorded for reflector-ISD and MS/MS spectra, respectively. Data were analyzed using Flex Analysis 3.0 and Bio-Tools 3.0 software (Bruker Daltonics, Bremen, Germany) taking into account the deduced amino acid sequence from ORF2 of PhV.

### Nucleic acid extraction

Viral ssRNA was extracted from purified virions, *P. halstedii *sporangia or herbarium specimens of *P. halstedii *infected sunflower using the Aurum Total RNA Mini Kit (Bio-Rad Laboratories, Hercules, CA) according to the manufacturer's protocols. RNA sample purity was checked spectroscopically (A_260 _/A_280 _ratio).

Viral dsRNA was extracted and purified from *P. halstedii *infected sunflower tissue [15].

### cDNA synthesis, primers, PCR and sequencing

cDNA synthesis was performed using either the First Strand cDNA Synthesis Kit or RevertAid First Strand cDNA Synthesis Kit (both kits: Fermentas, Glen Burnie, MD). A first fragment of RNA2 was obtained as stated earlier [1].

PhV specific primers used for sequence comparison were summarized in Table 2.

**Table 2:** Primers specifically designed for *Plasmopara halstedii *virus (PhV) which were used for sequence comparison.

PhV forward primers (5'→3')			
	RNA1 primer sequence		RNA2 primer sequence
1-f3	ATT TAC CGT GTT GCT GGA GG	2-f3	CTG GGT AGT GGA GAC TAC ACA
1-f12	CAC TAA CTA ACG CTT TCT GTG CT	2-f22	ACG CAG GAA AAC GAG GAA G
1-f34	GCC AGG GAT GTT GGT AGA GA	2-f28	ATT GTC CAA CGT AGC CTT CG
1-f39	TAC CAC AAT CGA AGG GTC AAG	2-f30	GCA ATC GCG TCG ACA AA
1-f42	CTT TCC GAC CTG AAT ACA CGA	2-f31	AGC GTG CCT ACT GAG GAT TC
1-f43	CCA CTG TGT GGC ACG ATT AC	2-f32	AGC CGG TGG ATC TGT AAA TG
1-f44	AAA GAG TGC TGG CGT TAC AGC	2-f33	GTT TTG GCG GAT TGG AAG TA
1-f45	GAC CAT TTC AAC CGG TAA GG		
			
**PhV reverse primers (5'→3')**			
	**RNA1 primer sequence**		**RNA2 primer sequence**

1-r17	GAA TTT GGA TAA AGC CCG AA	2-f3rc	TGT GTA GTC TCC ACT ACC CAG
1-r24	TAG GGA CGC TAA AGC AGC AT	2-r4	CGA GAC AGT TGC GTT GGA
1-r25	TAT TTG GTG GTC TGC ATC CA	2-r18	CAG TGG AAC GGT ATG ACG TG
1-r48	ATA GCA GTC AAT CCC GCA CT	2-r24	GTC TCC CCC AAC CAT TAT GA
		2-r27	TAT TGT GCA AAC CCA CTC GA

The PCR standard protocol was 94°C for 2 min; then 35 cycles of 94°C for 45 s, 54°C for 45 s, 72°C for 1 min 15 s; and a finale elongation step at 72°C for 10 min. Occasionally, a gradient thermal cycler set at different annealing temperatures was used to optimize the yield of PCR products.

PCR products were analyzed on 1% agarose gels (1× TBE buffer, ethidium bromide staining) and then purified (Qiaquick PCR Purification Kit; Qiagen, Valencia, CA). The purified PCR products were either directly sequenced with virus-specific primers or cloned in *E. coli *(StrataClone PCR U/A Cloning Kit, Stratagene, La Jolla, CA), isolated (GeneJet Plasmid Miniprep Kit; Fermentas, Glen Burnie, MD) and sequenced with standard primers. All sequences were at least once verified with another primer.

Using a gradient thermal cylcer, a primer set for RNA2 (2-r7: 5'-AAG CGC GGC GTT TGT -3' and 2-f9: 5'-CAA AGC GTC TCC CAT TGG - 3') resulted in two additional DNA fragments (0.8 kb and 0.7 kb, respectively) at an annealing temperature of 48°C. These two additional amplicons were directly sequenced. Their deduced amino acid sequences showed similarity to the deduced amino acid sequence of the RdRp of SmV A. Based upon this similarity, the sequence data of the putative PhV RNA1 were aligned and virus-specific primers were designed to close the gap between these two partial nucleotide sequences.

### 3' Rapid amplification of cDNA ends (3' RACE)

The 3' ends of RNA1 and RNA2 were determined when viral RNA was transcribed into cDNA with an adaptor (5'-ATG ACT CGA GTC GAC ATC GAT TTT TTT TTT TTT TTT TTT TTT TTT TTV N -3') which included a poly(T) tract (adaptor and adaptor-specific primer modified after Sambrook and Russell, 2001). After its synthesis, cDNA was amplified in PCR with virus-specific forward primers and an adaptor-specific reverse primer (5'-ATG ACT CGA GTC GAC ATC GA -3'). PCR products were cloned in *E. coli *and sequenced with virus-specific forward primers. Control experiments with *E. coli *poly(A) polymerase (NewEngland Biolabs, Ipswich, MA) indicated the lack of internal annealing sites for poly(T), thus confirming that both viral RNA segments featured a poly(A) tract at their 3' ends.

### 5' Rapid amplification of cDNA ends (5' RACE)

The 5' ends were determined using the SMART RACE cDNA Amplification Kit (Clontech Laboratories, Carlsbad, CA).

A second 5' RACE technique based on purified viral dsRNA was carried out [16] in order to ascertain the 5' ends of RNA1 and RNA2. The results of the second 5' RACE experiments confirmed the 5' sequence of the RNA1 segment. For the RNA2 segment, the 5' end determined with the SMART RACE-kit was extended by additional 44 nt.

### Comparative sequence analysis

Viral RNA of different *P. halstedii *isolates was transcribed into cDNA using random hexamer primers. PCR was then conducted using primer sets for RNA1 and RNA2, respectively. After submitting the PCR product to agarose gel electrophoresis, a single DNA fragment resulted for each primer pair. These fragments were purified and directly sequenced using one primer of the corresponding primer pair for sequencing. Both strands were sequenced at least twice.

### Sequence analysis

Sequence data were subjected to BLAST analysis and aligned using the software BioEdit (version 7.0.5.3; Hall, 1999). The GenBank accession numbers for the different PhV isolates are given in Table 1. The GenBank accession numbers for SmV A are AB083060 (RNA1), AB083061 (RNA2) and AB083062 (RNA3).

## Results and discussion

### Genome organization

Based on the recent comparison of a partial nucleotide sequence of PhV in various samples of *P. halstedii *[1], the viral isolate Ph8-99 was selected for complete sequencing of the two ssRNA segments and genome analysis.

RNA1 consisted of 2793 nt excluding the 3' poly(A) tract. Analysis of the determined nucleotide sequence revealed the existence of one large ORF (ORF1) of 2745 nt (914 amino acid residues). The estimated molecular mass of the protein encoded by ORF1 was 104 kDa. RNA1 had a 5'-untranslated region (5' UTR) of 18 nt and a 3'-untranslated region (3' UTR) of 30 nt. Analysis of the deduced amino acid sequence of ORF1 revealed the presence of the putative RdRp domain containing the GDD motif.

RNA2 consisted of 1526 nt excluding the 3' poly(A) tract. Analysis of the determined nucleotide sequence revealed the existence of one ORF (ORF2) of 1128 nt. The estimated molecular mass of the protein encoded by ORF2 was 40 kDa (375 amino acid residues). RNA2 had a 5' UTR of 164 nt and a 3' UTR of 234 nt. A sequence comparison with data of SmV A [3] and other sequences indicated that ORF 2 encoded for the viral CP of PhV.

### Coat protein (CP) characterization

**Figure 1:**
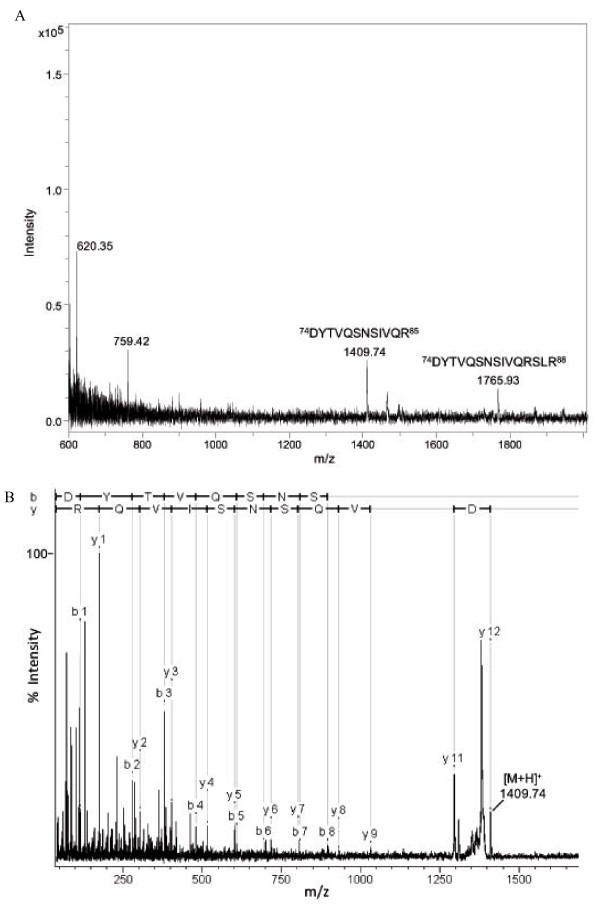
**Mass spectroscopic analysis of the coat protein (CP) of *Plasmopara halstedii *virus (PhV)**. **A **Reflector ISD-MALDI-TOF spectrum. The N-terminal sequence of the intact PhV CP was analyzed by Top-Down MALDI-TOF mass spectrometry. The in source decay (ISD) spectrum shows two major ISD fragment ions of the PhV CP with masses of 1409.74 Da and 1765.93 Da, respectively. These ISD fragment ions masses fit to sequences D74-R85 and D74-R88 of the deduced amino acid sequence of the PhV CP, respectively. The corresponding amino acid sequences of the ISD fragment ions were shown. **B **MS/MS spectrum of the 1409.74 Da ISD-fragment. The N-terminal sequence of PhV CP DYTVQSNSIVQR deduced from the reflector ISD spectrum (Fig. 1A) was confirmed by MS/MS analysis of the 1409.74 Da ISD fragment ion. The observed b- and y-fragment ions are annotated in the spectrum and the corresponding amino acid sequence covered by b- and y-ions were shown.

A molecular mass of the CP of ca. 37.5 kDa [8] to 36 kDa [1] was estimated previously by SDS-PAGE analysis. This was in discrepancy with the molecular mass of 40 kDa calculated for the 375 amino acids deduced from the nucleotide sequence of ORF2. Therefore, the CP band was cut off from an SDS-PAGE-gel, digested with trypsin and analyzed by mass spectrometry. Nano-LC-ESI-MS/MS analysis showed sequence coverage of 62% according to the deduced amino acid sequence from ORF2. The first 23 amino acids of the N-terminal sequence of CP were not covered by tryptic peptides and the peptide most proximal to the N-terminus of CP comprising the amino acids 24-35 (DYTVQSNSIVQR), had a non-tryptic cleavage site at its N-terminus (data not shown). This suggested that the N-terminus of the CP might be proteolytically processed *in vivo *leading to the lower molecular mass as observed in SDS-PAGE experiments. In order to verify this, the N-terminal sequence of the CP was analyzed by Top-Down MALDI-TOF analysis [17]. Two major in source decay (ISD) fragments of 1479.74 Da and 1765.93 Da were observed by Top-Down MALDI-TOF analysis (Figure 1A). Fragmentation of the smaller ISD fragment revealed the peptide sequence DYTVQSNSIVQR (Figure 1B), whereas the larger ISD fragment covered the sequence DYTVQSNSIVQRSLR (data not shown). Therefore, the Top-Down MALDI-TOF sequence analysis determined the start of the N-terminal sequence of CP at amino acid 24 and confirmed the result from the nano-LC-ESI-MS/MS experiment.

These results suggest that the CP of PhV is processed *in vivo *at its N-Terminus by a yet unknown protease to a final size of 352 amino acids and a mass of 38.0 kDa which is in consistency with the results obtained by SDS-PAGE analysis. Similar processing of the N-terminus was assumed for CP2 of SmV A [3].

### Viral nucleotide sequence comparison among different isolates of *P. Halstedii*

Variation in the nucleotide sequences of PhV from *P. halstedii *isolates of different pathogenicity and origin was assessed using virus-specific primers for large fragments of both RNA segments.

Seven representative *P. halstedii *virus isolates (Ph4-93, Ph1-97, Ph9-98, Ph1-00, Ph10-00, Ph19-01 and Ph5-05) were chosen to be partially sequenced (Table 1). A 1.4 kb-sequence of RNA2 and a 1.9 kb-sequence of RNA1 were compared to the complete sequence of *P. halstedii *virus isolate Ph8-99 and compared among themselves. Accounting for more than three quarters of the total genome of *P. halstedii *virus, sequence variation was extremely low.

In terms of RNA1, the two French isolates (Ph8-99 and Ph1-00) were identical. The samples from Germany (Ph9-98), Hungary (Ph19-01), and the USA (Ph4-93) each differed from isolate Ph8-99 in single nucleotides at different positions. Only the sequence variation in the German sample led to an amino acid exchange in the deduced amino acid sequence.

In terms of RNA2, Ph8-99 differed in a single nucleotide from all other samples causing an amino acid exchange from alanine to valine. The Hungarian isolate Ph19-01 differed from isolate Ph8-99 in one nucleotide which led to an amino acid exchange from isoleucine to methionine. The French isolate Ph1-00 differed from Ph8-99 in two nucleotides causing a single amino acid exchange from glutamine to serine. The German isolate Ph5-05 showed one nucleotide insertion in the 3'-UTR and additionally one nucleotide exchange. The US isolate Ph4-93 showed only the nucleotide insertion like the German isolate Ph5-05 but not the additional nucleotide exchange. Since the insertion took place in the 3'-UTR, the frameshift was without consequences for the structure or function of the CP.

It appeared that alterations in these highly conserved sequences of RNA1 and RNA2 are detrimental for the self-assembly process during virus propagation.

Additionally five infected herbarium specimens of sunflower were tested to fathom the applicability of PhV sequence population studies on a broader set of samples. The herbarium specimens were stored in the herbarium of the University of Hohenheim for up to nine years. Small fragments between 410 and 650 nt of RNA2 were amplified and sequenced. Again, the sequence variation of the virus in these herbarium specimens was insignificant (data not shown).

The results of this study suggested the presence of a single virus in the tested *P. halstedii *isolates.

### Comparison of PhV with other viruses

A preliminary study revealed that PhV and SmV A shared several morphological, biochemical and molecular characteristics. Both viruses were isometric and showed a granular surface. PhV measured ca. 37 nm in diameter, whereas SmV A was slightly larger (ca. 40 nm in diameter). Both viral genomes are encoded in ssRNA. In SmV A, three RNA segments were detected whereas PhV only contained two segments [1,3-5]. The genome organization was now studied and compared to other viruses.

SmV A RNA1 with 2928 nt coded for the RdRp on ORF1a and another protein of unknown function in ORF1b. In PhV, RNA1 with its 2793 nt (excluding the 3' poly(A) tract) coded analogously for the RdRp. ORF1b as it was found in SmV A was not determined in PhV RNA1 (Table 3).

**Table 3:** Comparison of *Plasmopara halstedii *virus (PhV) and *Sclerophthora macrospora *virus A (SmV A).

	PhV	SmV A
Morphology		
Virion diameter (stained with 2% uranyl acetate)	37 nm	40 nm
Virion shape	isometric	isometric
Presence or absence of peplomers	present	present
**Genome**		

Type of nucleic acid	RNA	RNA
Strandedness	single-stranded	single-stranded
Size of genome	4.3 kb	5.9 kb
Number of segments	2	3
G+C ratio	RNA1: 45% RNA2: 48% -	RNA1: 51% RNA2: 49% RNA3: 51%
Size of segments and open-reading frames (ORF)	RNA1: 2793 nt (ORF1) RNA2: 1526 nt (ORF2) -	RNA1: 2928 nt (ORF1a, ORF1b) RNA2: 1981 nt (ORF2) RNA3: 977 nt (no ORF)
Size and function of ORF RdRp: RNA-dependent RNA polymerase CP: Coat protein	ORF1: 2745 nt (RdRp) - ORF2: 1128 nt (CP)	ORF1a: 2697 nt (RdRp) ORF1b: 870 nt (unknown function) ORF2: 1269 nt (CP)
Size of 5' untranslated region (5' UTR)	RNA1: 18 nt RNA2: 164 nt	RNA1: 66 nt RNA2: 11 nt
Size of 3' untranslated region (3' UTR)	RNA1: 30 nt RNA2: 234 nt	RNA1: 165 nt RNA2: 701 nt
3'-terminal poly(A) tract	Poly(A) tracts are present in RNA1 and RNA2.	Poly(A) tracts are lacking at the 3' ends of all 3 RNA.
**Proteins**		

Number of structural proteins	1	2
Size of structural proteins	SDS-PAGE: 36 kDa ORF2: 40 kDa (375 amino acids) Postproteolytic cleavage: 38 kDa	SDS-PAGE: 43 kDa and 39 kDa ORF2: 45 kDa (422 amino acids) Postproteolytic cleavage: 38 kDa
CP shows similarities to CP of...	SmV A, Tombusviruses	PhV, Tombusviruses
Number of non-structural proteins	1	1-2
Size of non-structural proteins	ORF1: 914 amino acids (104 kDa) -	ORF1a: 898 amino acids (100 kDa) ORF1b: 289 amino acids (33 kDa)
RdRp shows similarities to RdRp of...	SmV A, Nodaviruses	PhV, Nodaviruses
**Lipids**		

Content, character, etc.	none	not determined

The deduced amino acid sequence of PhV ORF1 (RdRp) showed similarity of ca. 47% to the deduced amino acid sequence of SmV A ORF1a (RdRp). Moreover, an amino acid sequence similarity of ca. 40% was observed between PhV RdRp and RdRp of viruses within the Nodaviridae family (*e.g*. Barfin flounder nervous necrosis virus, Striped Jack nervous necrosis virus).

RNA2 of SmV A with a length of 1981 nt coded for the two CP. In PhV, RNA2 of 1526 nt (excluding the 3' poly(A) tract) was found to code for the single CP (Table 3). Between ORF2 of PhV and SmV A ORF2, amino acid sequence similarity of ca. 56% was observed. Within the first ten amino acids at the N-terminal sequences, PhV CP (DYTVQSNSIV) and CP2 of SmV A (DYKVSQNSLV) featured six identical amino acid residues. Two other amino acid QS (PhV) and SQ (SmV A), respectively, were interchanged. Amino acid sequence similarity of ca. 37% was observed between the CP of PhV and the CP of viruses within the Tombusviridae (*e.g*. Pelargonium leaf curl virus, Tomato bushy stunt virus).

In PhV, RNA1 and RNA2 both had poly(A) tracts at their 3' termini whereas in SmV A poly(A) tracts were lacking at the 3'-termini of all three viral RNA (Table 3).

## Conclusions

The complete nucleotide sequence of PhV was established. PhV showed similarities to SmV A and viruses within the Tombusviridae family as well as Nodaviridae family.

The sequence data of several viral isolates suggested that there was a single virus type in different *P. halstedii *isolates. Viral sequence variation did not account for different pathotypes of the oomycete *P. halstedii*.

## Competing interests

The authors declare that they have no competing interests.

## Authors' contributions

MHD carried out most of the experiments, analyzed the sequences and drafted the manuscript. JCG participated in designing the experiments, extracted viral dsRNA and coat protein and carried out further experiments. JP analyzed the viral coat protein and prepared the respective portion of the manuscript. OS coordinated this study, assisted sequence analysis and helped preparing the manuscript.

All authors have read and approved the final manuscript.
